# Acute Hemichorea Can Be the Only Clinical Manifestation of Post-Varicella Vasculopathy: Two Pediatric Clinical Cases

**DOI:** 10.3389/fneur.2018.00164

**Published:** 2018-03-20

**Authors:** Chiara Davico, Carlotta Canavese, Aba Tocchet, Chiara Brusa, Benedetto Vitiello

**Affiliations:** ^1^Division of Child and Adolescent Neuropsychiatry, University of Turin, Turin, Italy

**Keywords:** hemichorea, varicella zoster virus, vasculopathy, children, brain MRI

## Abstract

Acute hemichorea can occur in the context of infectious, autoimmune, metabolic, toxic, and vascular neuropathologies. Primary infection by varicella zoster virus (VZV) can result in vasculopathy with neurological manifestations, such as hemiparesis, at times accompanied by hemichorea. Isolated hemichorea, however, had not been reported. We here describe two cases of VZV-induced vasculopathy whose sole clinical manifestation was acute hemichorea. Both cases involved young boys of 3 years of age, who presented with acute hemichorea 4–6 months after initial VZV infection. All hematological, immunological, and toxicological tests were normal, except for the presence of VZV IgG. Brain structural magnetic resonance imaging (MRI) and magnetic resonance angiography revealed specific signs of vasculitis and ischemic lesions in the basal ganglia region (lentiform nucleus, thalamus, and internal capsule). Following corticosteroid and acetylsalicylic acid treatment, full symptomatic recovery was achieved within 3 weeks. Repeated MRI documented full neurostructural recovery, which was confirmed at extended follow-up for more than 1 year. These cases indicate that VZV-induced vasculopathy should be considered in the case of pediatric isolated acute hemichorea.

## Background

Hemichorea is a rather uncommon clinical presentation that results from unilateral basal ganglia damage (1). Its etiopathogenesis can be diverse, including infectious, autoimmune, metabolic, toxic, and vascular mechanisms (2–6). In school-age children, hemichorea is often a manifestation of Sydenham’s chorea, even though bilateral chorea is the usual presentation (2). A well-known mechanism underlying choreic movements is vasculopathy leading to hemorrhagic or hypoxic–ischemic lesions of the basal ganglia (7).

The varicella zoster virus (VZV) is a highly contagious neurotropic alpha-herpes virus, whose primary infection results in a typical exanthematic disease (varicella) and can be complicated by vasculopathy. VZV-induced vasculopathy can lead to ischemic or hemorrhagic lesions, with possible clinical manifestations of stroke, transient ischemic attacks, and hemiparesis, with or without hemichorea (8, 9). It is estimated that VZV vasculopathy accounts for about one-third of all pediatric strokes, usually presenting as acute hemiparesis (7, 10–12). However, to the best of our knowledge, no case of acute hemichorea with MRI-documented evidence of basal ganglia and internal capsule ischemic lesions after VZV infection had been reported. We here describe two clinical cases of VZV vasculopathy in young children whose sole clinical sign was acute hemichorea.

## Case 1

**Figure 1:**
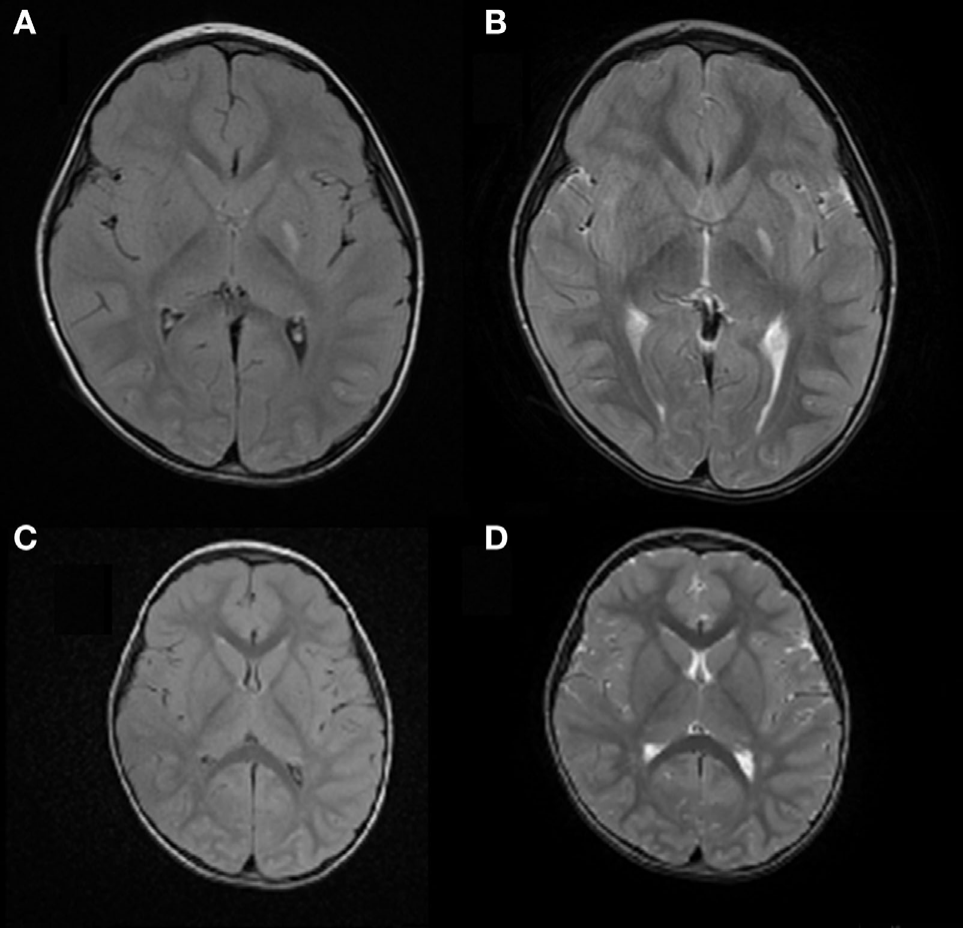
Case 1: **(A)** axial FLAIR image and **(B)** axial T2 weighted image, showing ischemic lesion involving the left lentiform nucleus and posterior limb of the internal capsule. **(C)** Axial FLAIR image and **(D)** axial T2 weighted image showing resolution of the left lentiform nucleus lesion and reduction of the posterior limb involvement in the internal capsule.

A 3-year and 8-month-old boy presented to the hospital emergency department for acute onset of involuntary movements of the right side of the body. Except for hemichorea, no other neurological sign or symptom was detected. Six months before the acute onset of hemichorea, he had had varicella (chickenpox). He was previously healthy, with negative family history of movement disorders. Complete blood count, erythrocyte sedimentation rate (ESR), C reactive protein (CRP), hemoglobin S, kidney, liver and thyroid function, anti-DNAse B antibodies, total immunoglobulines, lupus anticoagulant, antinuclear antibodies, anticardiolipin antibodies, anti-B2 glycoprotein antibodies, and coagulation tests were normal. Toxicological screening and metabolic panel were negative. Pharyngeal swab was normal, as well as electrocardiogram (ECG) and electroencephalogram (EEG). Serum anti-VZV IgGs were positive. Brain MRI (Figures 1A,B) revealed ischemic lesions involving the left lentiform nucleus and the posterior limb of internal capsule.

Following treatment with acetylsalicylic acid (2 mg/kg/day), prednisone (0.9 mg/kg/day), and haloperidol (0.03 mg/kg/day), there was a complete resolution of the hemichorea by 3 weeks after the acute onset. Four months later, brain MRI showed resolution of the lesion in the left lentiform nucleus and a reduction of the lesion in the posterior limb of the internal capsule (Figures 1C,D). An extended follow-up for 16 months confirmed the complete clinical recovery.

## Case 2

**Figure 2:**
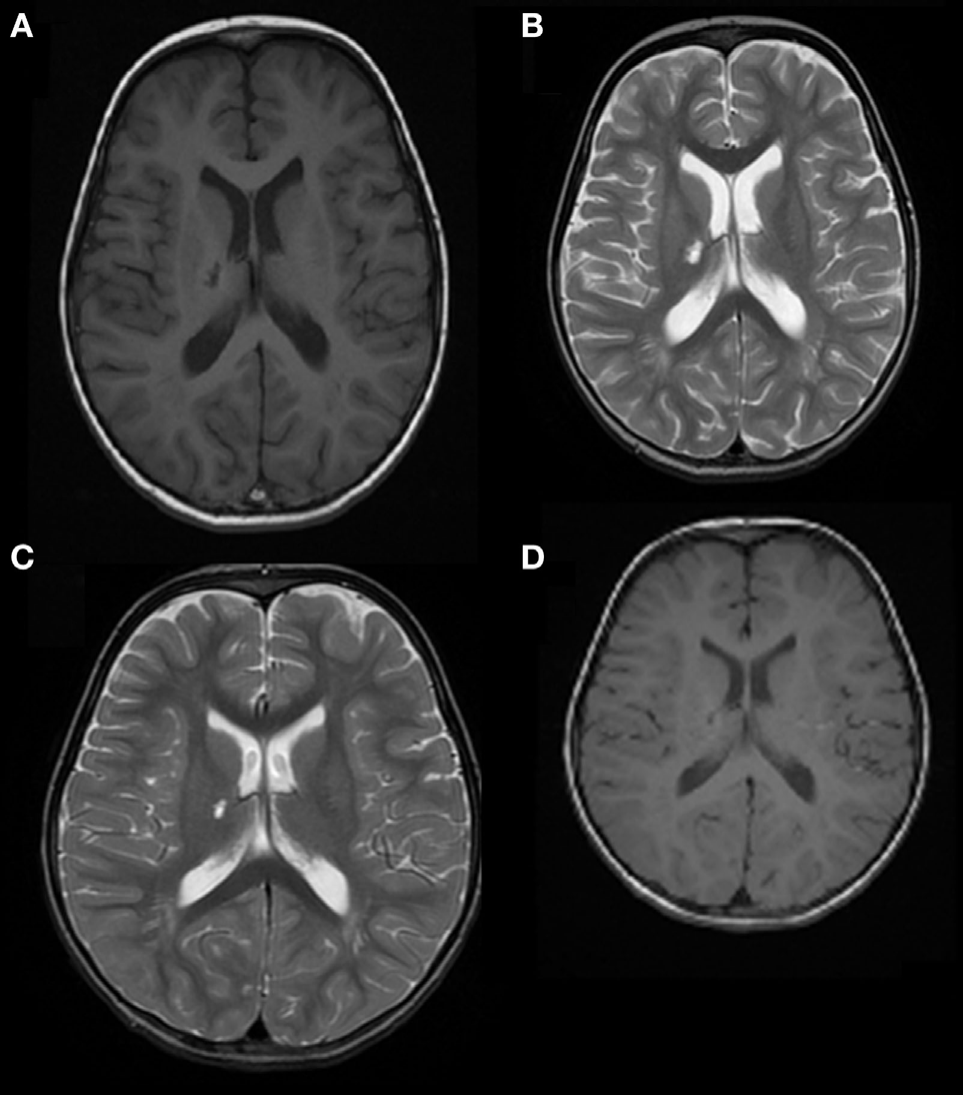
Case 2: **(A)** axial T1 image and **(B)** axial T2 weighted image, showing right thalamic nucleus and posterior limb of the internal capsule ischemic lesions. **(C)** Axial T2 weighted image and **(D)** axial T1 weighted image showing resolution of the right thalamic nucleus lesion and reduction of the involvement of the posterior limb of the internal capsule.

**Figure 3:**
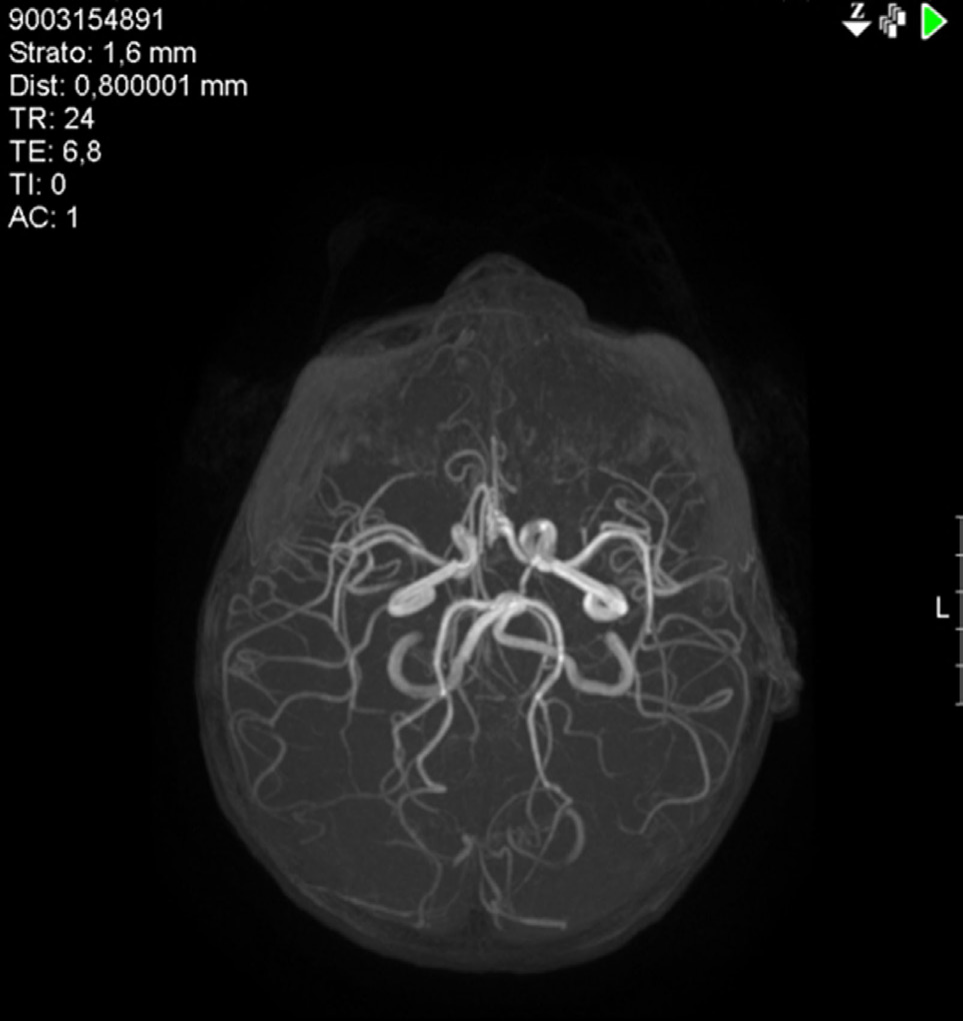
Case 2: Magnetic resonance angiography (MRA) showing reduced caliber of the right internal carotid artery, especially the supra-clinoid segment, and of the M1 segment of the middle cerebral artery, hypoplasia of the right A1 segment of the anterior cerebral artery, and tortuosity of the vertebrobasilar system.

A 3-year and 5-month-old boy was brought to the hospital emergency department with left side hemichorea that had suddenly appeared about 2 days earlier. No other neurological sign was detected. Medical history was significant for varicella 4 months before the admission. Family history was negative for movement disorders. Laboratory investigations, including blood count, ESR, CRP, hemoglobin S, kidney, liver and thyroid function, anti-DNAse B antibodies, total immunoglobulines, lupus anticoagulant, antinuclear antibodies, anticardiolipin antibodies, anti-B2 glycoprotein antibodies, and extensive coagulation tests, were all normal. Serum VSZ IgGs were positive. Metabolic panel, EEG, ECG, and echocardiogram were normal. Brain MRI revealed ischemic lesions in the right thalamic nucleus and the posterior limb of internal capsule (Figures 2A,B). Magnetic resonance angiography (MRA) showed a reduced caliber of right internal carotid artery, especially evident in the supra-clinoid segment, and of the M1 segment of the middle cerebral artery, hypoplasia of the right A1 segment anterior cerebral artery and tortuosity of the vertebrobasilar system (Figure 3).

Treatment with acetylsalicylic acid (2 mg/kg/day), prednisone (0.4 mg/kg/day), and haloperidol (0.03 mg/kg/day) was initiated. The hemichorea immediately improved, and the boy recovered completely in 3 weeks. Five months later, a brain MRI (Figures 2C,D) showed resolution of the lesion in the right thalamic nucleus and a reduction of the posterior limb of internal capsule involvement. This picture was consistent with a post-VZV vasculitis causing an asymptomatic ischemic lacunar lesion, and a transient hypoperfusion leading to hemichorea. An extended follow-up for 28 months confirmed the complete clinical recovery.

## Discussion

Acute hemichorea is due to a contralateral damage of the basal ganglia. The etiopathogenesis can involve infectious, autoimmune, vascular, metabolic, or toxic mechanisms, leading to acute injury of the putamen and/or caudate nucleus (1, 13). The most common cause in children is rheumatic fever, an autoimmune reaction triggered by group A beta-hemolytic *Streptococcus* infection that can manifest with abnormal, involuntary movements (Sydenham’s chorea). The typical neurological manifestation of rheumatic fever, however, is bilateral chorea, rather than hemichorea (2). The age of onset of Sydenham’s chorea is usually 8–9 years. In children, hemichorea can also be one of the signs of Rasmussen’s encephalitis, a rare and progressive autoimmune unilateral inflammation of the brain that results in seizures and hemiparesis (13).

Chorea and, less frequently, hemichorea have been reported also in the context of viral infections. VZV, herpes simplex, and influenza A viruses can cause encephalitis with various movement disorders, including also chorea (4, 14, 15). HIV infection can set the stage for opportunistic infections, such as histoplasmosis, with central nervous system involvement and consequent hemichorea (16).

To the best of our knowledge, this is the first report of acute-onset hemichorea as the sole neurological sign of a recent VZV infection. These otherwise healthy 3-year-old children suddenly developed hemichorea 4 and 6 months, respectively, after the primary VZV infection. Other possible etiological explanations were excluded, including Sydenham’s chorea, which is rare under 5 years of age (17). Extensive hematological, endocrine, autoimmune, and ECG and EEG assessments were conducted. CSF was not obtained because it was not clinically indicated. It should be noted that testing for VZV IgG in the CSF may not exclude a diagnosis of VZV-induced vasculopathy because of the low sensitivity in children (18). The time interval of 4–6 months, from the primary infection until the appearance of hemichorea, is consistent with previous reports of VZV vasculopathy (19). Finally, repeated MRI examinations documented vasculopathic lesions of the basal ganglia area, which resolved in the following weeks together with the hemichorea. It has been previously documented that the typical brain MRI signs of VZV vasculopathy are ischemic lesions of the basal ganglia and/or the internal capsule (19).

The most common complications of varicella are bacterial infections of the skin and soft tissues in children and pneumonia in adults. Less frequent are viral pneumonia, septicemia, toxic shock, necrotizing fasciitis, osteomyelitis, septic arthritis, and neurological complications. The neurotropism of VZV is well known. After the primary infection, VZV usually establishes in the neurons of the cranial nerves, dorsal roots, and autonomic ganglia, as well as the adrenal glands, remaining latent for many years before reactivating in debilitated and immunocompromised individuals to cause herpes zoster (shingles). Neurological involvement can appear also during the primary VZV infection or in the following 4–6 months (but cases as early as 1 week and as long as 48 months have been reported). In children, rates of post-VZV neurological complications ranging from 3.9 to 61.3% have been reported (4, 20, 21). Severe neurological complications, however, are rare in immunocompetent children (4). Central nervous system involvement may cause encephalitis, meningitis, cerebellitis, acute myelitis, and vasculopathy, with various possible clinical manifestations, such as stroke or stroke-like episodes, ataxia, seizures, acute headache, cranial nerve palsy, syncope, tremors, and drowsiness.

VZV-induced angiopathy is much more common after the initial infection than with herpes zoster. It can involve different kind of vessels: central or peripheral, and arteries or veins of different caliber. For example, deep venous thrombosis can lead to pulmonary embolism or purpura fulminans, as well as arterial complications, such as giant cells arteritis affecting extracranial circulation or granulomatous aortitis (19, 22). VZV can affect intracerebral arteries, producing changes in arterial caliber and contractility and leading to stroke. The underlying pathogenesis of VZV vasculopathy is probably mediated by accumulation of inflammatory cells in the media layer of the cerebral arteries, with progressive thickening of the vessel wall and dysfunction in nitric oxide metabolism (19).

The prognosis of the neurological complications of VZV vasculopathy is usually good, with complete functional recovery, even without specific pharmacological treatment (7). However, residual symptoms, such as dystonia, have been reported at a rate as high as 12% (10, 23). The efficacy of treatment in VZV vasculopathy is controversial, and there is debate about the benefit of antiviral and anti-inflammatory therapy. Acyclovir is recommended in cases in which VZV is isolated in the CSF (24). Both salicylates and corticosteroids have been used in an attempt to ameliorate the inflammatory processes, and antidopaminergic agents, such as haloperidol, can help control the movement disorder when this is distressing or invalidating. However, the efficacy of these treatments for accelerating or achieving recovery is unclear.

In conclusion, these two cases provide evidence that acute hemichorea can be the only clinical manifestation of post-VZV vasculopathy. VZV vasculopathy should be considered among the possible causes of acute-onset hemichorea, especially in children younger than 5 years, even in the absence of hemiparesis.

## Ethics Statement

Written informed consent to publish this report was obtained from the parents of the children.

## Author Contributions

CD, CC, AT, and CB: clinical data collection and interpretation of laboratory and brain imaging results and manuscript preparation. BV: data interpretation, critical review, and manuscript preparation.

## Conflict of Interest Statement

The authors declare that the research was conducted in the absence of any commercial or financial relationships that could be construed as a potential conflict of interest.

## References

[1] Laganiere S Boes AD Fox MD. Network localization of hemichorea-hemiballismus. Neurology (2016) 86:2187–95. 10.1212/WNL.0000000000002741 27170566 PMC4898318

[2] Cardoso F. Autoimmune choreas. J Neurol Neurosurg Psychiatry (2017) 88:412–7. 10.1136/jnnp-2016-314475 27919056

[3] Isaacs D Cmelak A Kirschner AN Phibbs F Radiotherapy-induced hemichorea. Neurology (2016) 86:1355–7. 10.1212/WNL.0000000000002546 26944270

[4] Bozzola E Tozzi AE Bozzola M Krzysztofiak A Valentini D Grandin A Neurological complications of varicella in childhood: case series and a systematic review of the literature. Vaccine (2012) 30:5785–90. 10.1016/j.vaccine.2012.05.057 22683522

[5] Battisti C Forte F Rubenni E Dotti MT Bartali A Gennari P Two cases of hemichorea-hemiballism with nonketotic hyperglycemia: a new point of view. Neurol Sci (2009) 30(3):179–83. 10.1007/s10072-009-0039-5 19305947

[6] Watanabe T Onda H. Hemichorea with antiphospholipid antibodies in a patient with lupus nephritis. Pediatr Nephrol (2004) 19:451–3. 10.1007/s00467-003-1388-6 14740286

[7] Ciccone S Faggioli R Calzolari F Sartori S Calderone M Borgna-Pignatti C. Stroke after varicella-zoster infection: report of a case and review of the literature. Pediatr Infect Dis J (2010) 29:864–7. 10.1097/INF.0b013e3181ddefb6 20803842

[8] Nagel MA Traktinskiy I Azarkh Y Kleinschmidt-DeMasters B Hedley-Whyte T Russman A Varicella zoster virus vasculopathy: analysis of virus-infected arteries. Neurology (2011) 77:364–70. 10.1212/WNL.0b013e3182267bfa 21753174 PMC3140801

[9] Borbinha C Marto JP Calado S Viana-Baptista M. A young woman with ischemic stroke: should we pay more attention to varicella zoster infection? Case Rep Neurol (2016) 8:145–50. 10.1159/000447296 27504091 PMC4965528

[10] Reis AF Pais P Monteiro JP. Chickenpox and stroke in children: case studies and literature review. Acta Paediatr (2014) 103:e176–80. 10.1111/apa.12535 24330378

[11] Miravet E Danchaivijitr N Basu H Saunders DE Ganesan V. Clinical and radiological features of childhood cerebral infarction following varicella zoster virus infection. Dev Med Child Neurol (2007) 49:417–22. 10.1111/j.1469-8749.2007.00417.x 17518925

[12] Askalan R Laughlin S Mayank S Chan A MacGregor D Andrew M Chickenpox and stroke in childhood: a study of frequency and causation. Stroke (2001) 32:1257–62. 10.1161/01.STR.32.6.1257 11387484

[13] Bekiesinska-Figatowska M Mierzewska H Jurkiewicz E. Basal ganglia lesions in children and adults. Eur J Radiol (2013) 82:837–49. 10.1016/j.ejrad.2012.12.006 23313708

[14] Hacohen Y Deiva K Pettingill P Waters P Siddiqui A Chretien P N-methyl-D-aspartate receptor antibodies in post-herpes simplex virus encephalitis neurological relapse. Mov Disord (2014) 29:90–6. 10.1002/mds.25626 24014096

[15] Ryan MM Procopies PG Ouvrier RA. Influenza A encephalitis with movement disorder. Pediatr Neurol (1999) 21:669–73. 10.1016/S0887-8994(99)00062-4 10513697

[16] Estrada-Bellmann I Camara-Lemarroy CR Flores-Cantu H Calderon-Hernandez HJ Villareal-Velazquez HJ. Hemichorea in a patient with HIV-associated central nervous system histoplasmosis. Int J STD AIDS (2016) 27:75–7. 10.1177/0956462414564608 25505048

[17] Tani LY Veasy LG Minich LL Shaddy RE. Rheumatic fever in children younger than 5 years: is the presentation different? Pediatrics (2003) 112:1065–8. 10.1542/peds.112.5.1065 14595047

[18] Bartolini L Gentilomo C Sartori S Calderone M Simioni P Laverda AM. Varicella and stroke in children: good outcome without steroids. Clin Appl Thromb Hemost (2011) 17:E127–30. 10.1177/1076029610389025 21159706

[19] Nagel MA Jones D Wyborny A. Varicella zoster virus vasculopathy: the expanding clinical spectrum and pathogenesis. J Neuroimmunol (2017) 308:112–7. 10.1016/j.jneuroim.2017.03.014 28335992 PMC5489071

[20] Ziebold C von Kries R Lang R Weigl J Schmitt HJ. Severe complications of varicella in previously healthy children in Germany: a 1-year survey. Pediatrics (2001) 108(5):E79. 11694663

[21] Almuneef M Memish ZA Balkhy HH Alotaibi B Helmy M. Chickenpox complications in Saudi Arabia: is it time for routine varicella vaccination? Int J Infect Dis (2006) 10:156–61. 10.1016/j.ijid.2005.02.008 16260166

[22] Driesen Y Verweij M De Maeseneer M De Dooy J Wojciechowski M Van Den Akker M. Vascular complications of varicella: description of 4 cases and a review of literature. Pediatr Infect Dis J (2015) 34:1256–9. 10.1097/INF.0000000000000855 26226447

[23] Rack AL Grote V Streng A Belohradsky BH Heinen F von Kries R Neurologic varicella complications before routine immunization in Germany. Pediatr Neurol (2010) 42:40–8. 10.1016/j.pediatrneurol.2009.07.012 20004861

[24] Gilden D Cohrs RJ Mahalingam R Nagel MA. Varicella zoster virus vasculopathies: diverse clinical manifestations, laboratory features, pathogenesis, and treatment. Lancet Neurol (2009) 8:731–40. 10.1016/S1474-4422(09)70134-6 19608099 PMC2814602

